# Acute Buried Bumper Syndrome: A Case Report

**DOI:** 10.7759/cureus.36289

**Published:** 2023-03-17

**Authors:** Lefika Bathobakae, Celia Leone, Mohamed M Elagami, Hardikkumar Shah, Walid Baddoura

**Affiliations:** 1 Internal Medicine, St. Joseph's Regional Medical Center, Paterson, USA; 2 Internal Medicine, St. George's University School of Medicine, St. Georgestown, GRD; 3 Gastroenterology and Hepatology, St. Joseph's Regional Medical Center, Paterson, USA

**Keywords:** enteral nutrition, complication, endoscopy, percutaneous endoscopic gastrostomy, buried bumper syndrome

## Abstract

Buried bumper syndrome (BBS) is a rare but severe complication of percutaneous endoscopic gastrostomy (PEG) tube feeding. Patients with BBS lose PEG tube patency and may experience peristomal pain, content leaks, or peritonitis. An early diagnosis can avert further complications. BBS is a clinical diagnosis, but an abdominal computerized tomography scan or upper endoscopy is needed to confirm the diagnosis. BBS is a long-term complication of PEG tube feeding, and cases of acute onset are scant in the literature. We report a unique case of a 65-year-old female with a history of stroke who developed BBS five weeks after PEG tube placement.

## Introduction

A percutaneous endoscopic gastrostomy (PEG) tube is a standard feeding modality for patients requiring long-term enteral feeding support [1,2], and its use has been increasing since the 1980s [3-5]. Buried bumper syndrome (BBS) is a rare but serious long-term complication of PEG tube feeding [6-8] and has a prevalence of 1.5% to 9% [2,6]. A PEG tube usually lasts up to 1-2 years, and tube degradation often indicates replacement [9,10]. On average, BBS develops eighteen months post-PEG tube placement [5], and acute cases of BBS are scarce in the literature. 

In BBS, an internal bolster of the PEG tube migrates along the stoma tract and gets lodged anywhere between the gastric mucosa and the abdominal wall [3,6,11-13]. The tight positioning of the external bumper is thought to cause a traction force, which displaces the internal bolster into the gastric wall. This traction force also causes tissue ischemia and necrosis, which may result in scarring or abscess formation [13].

BBS can be classified as incomplete or complete depending on the positioning of the displaced internal bumper [8]. In incomplete BBS, the internal bolster lodges in the gastric wall [8] and can be seen on upper endoscopy. In complete BBS, however, the dislodged bumper buries deep into the gastric wall and cannot be visualized on endoscopy [8]. A PEG tube material, tube size, or distance of the external bumper from the abdominal wall can influence the incidence of BBS in PEG-feeding patients [3]. Other risk factors for BBS include weight gain after PEG tube insertion, advanced age, obesity, and chronic cough. We herein present a rare case of a patient who developed BBS five weeks after PEG tube placement.

## Case presentation

A 65-year-old female with a past medical history of insulin-dependent diabetes mellitus, dyslipidemia, hypertension, and prior left cerebellar infarct presented to the emergency department (ED) for evaluation of unsteady gait and right-sided weakness with aphasia. The patient was admitted to the intensive care unit for acute left pontine stroke and later transferred to the neurological unit for further management. Her hospital course was complicated by new-onset atrial fibrillation and oropharyngeal dysphagia. After a month in the hospital, the patient underwent a PEG tube placement for long-term enteral feeding and medications.

The esophagogastroduodenoscopy (EGD) revealed two non-bleeding small gastric ulcers and erythematous duodenopathy. A relook endoscopy confirmed the correct positioning of the gastrostomy tube. The final tension and compression of the abdominal wall by the PEG tube and external bumper were checked and revealed that the bumper was loose, and lightly touching the skin. The external bolster was 1 cm from the abdominal wall. Five weeks after tube placement, a nurse reported a loss of PEG tube patency, and the gastroenterology service was consulted for assessment.

On assessment at the bedside, the patient was aphasic but in no acute distress. Her vital signs were stable. The PEG tube suctioning confirmed the pink discoloration of the aspirate. On physical exam, the patient had an obese and soft abdomen with normoactive bowel sounds. A PEG tube was in place but immobile. There was minimal dry blood around the PEG tube and mild tenderness to palpation around the PEG site with a palpable bumper. There was no rebound tenderness, guarding, or rigidity. The patient had a normal respiratory effort, and her lungs were clear to auscultation throughout. She was drowsy but easily arousable. We held the PEG tube feeds and started the patient on Pantoprazole 40 mg intravenously twice daily. We also stopped the heparin to lower the risk of bleeding.

**Figure 1:**
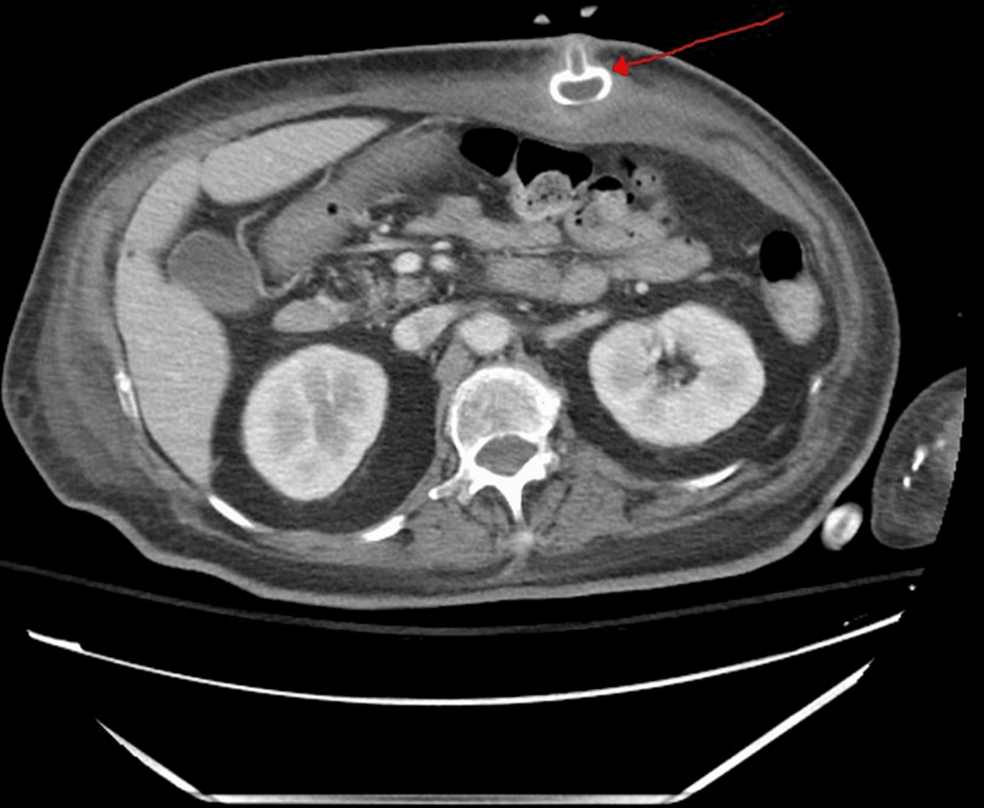
Computerized tomography scan of the abdomen and pelvis with contrast showing the PEG tube internal bolster buried in the gastric wall. An axial view of the CT scan of the abdomen and pelvis demonstrating the displacement of the PEG tube internal bolster into the gastric wall. The internal bumper appears extrinsic to the stomach, impinging on the gastric wall but not within the lumen.

The complete blood count and serum electrolyte levels were unremarkable. A computer tomography (CT) scan of the abdomen and pelvis with contrast (Figure 1) confirmed the BBS. On the CT scan, the PEG tube appeared extrinsic to the stomach, impinging on the gastric wall but not within the lumen.

**Figure 2:**
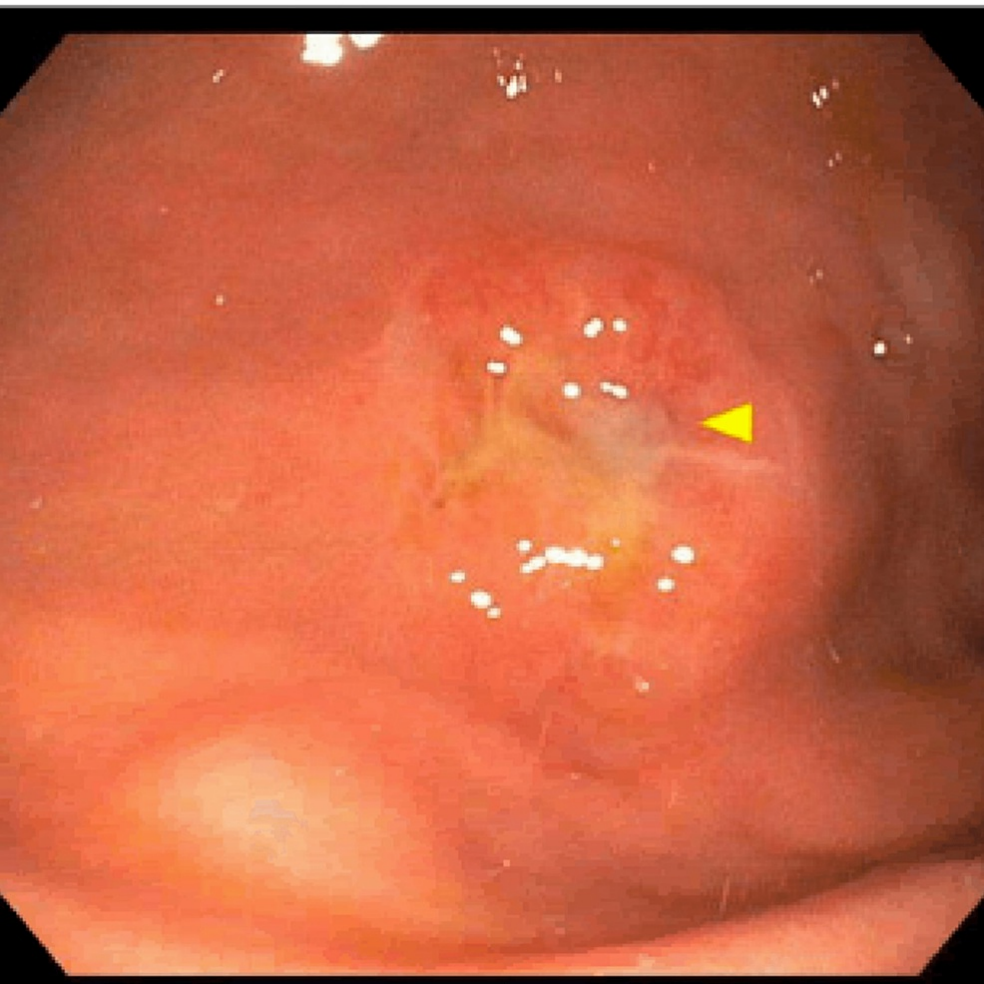
An endoscopic image showing a gastric body scar due to a buried bumper.

We removed the PEG tube externally with simple traction. We maintained the patient on intravenous fluids for a day and replaced the PEG tube the next day. Because of a narrowed stoma tract and scarring (Figure 2), the replacement PEG was inserted at a new site. It has been six months since we replaced the PEG tube, and it continues to function well for enteral feeds and medication administration.

## Discussion

A PEG tube is a reliable feeding modality for patients requiring long-term enteral feeding support [1,2]. Gaunderer and Ponsky introduced this intervention in the 1980s as a feeding alternative for patients with oropharyngeal dysphagia [1]. Other indications for gastrostomy tube feeding include severe bowel motility disorders, severe facial trauma, neurological disorders, head and neck cancers [8,11], burns, and short-bowel syndrome [1,5,11].

Rarely, a PEG may be used for gastropexy [6]. Children with malabsorption disorders from cystic fibrosis or tracheoesophageal fistulas also benefit from PEG feeding [13]. A PEG tube use is relatively contraindicated in patients with esophageal cancers due to concern for seeding the PEG tract with cancer cells [13]. In rare instances, a Russell introducer “push” technique may be used for head and neck cancers due to the lower risk of hematogenous spread of the cancer cells. PEG tube use is also highly discouraged in patients with bleeding disorders, massive ascites, refractory gastroparesis, sepsis, or hemodynamic instability [13].

Much like any other medical procedure, a PEG tube use may result in complications. The early complications are usually minor and tend to occur during or immediately after the procedure. These include tube dislodgement or dysfunction, aspiration, metabolic derangements, diarrhea, or infection [3,13]. Some patients may experience PEG site pain or skin maceration due to content leakages [1,8,11]. Peristomal wound infections are a common complication of PEG use and usually resolve with antibiotic therapy [8]. Antibiotic prophylaxis during PEG placement has led to a lower incidence of PEG site infections [8].

Significant complications of PEG tube use are uncommon and have been reported in 0.4% to 4.4% of the procedures [8]. These may include peristomal leakage with peritonitis, severe sepsis, post PEG fistula, gastro-enteric or gastro-colonic fistula, necrotizing fasciitis of the anterior abdominal wall, gastric bleeding [1,8,11], tumor seeding at the PEG site, BBS, or death. Some case reports have also reported internal organ perforation after PEG placement [4]. Proper techniques for transillumination and abdominal palpation may avert visceral organ perforation [4].

BBS is a rare but serious complication of PEG tube feeding [1,3,6]. The median period for the development of BBS is 18 months [5]. Even so, isolated cases of BBS developing acutely have been reported in the literature. Advanced age, obesity, and weight gain after the PEG placement are associated with a higher incidence of BBS. An excessive traction force between the internal bolster and external bumper is thought to cause this condition [8,12]. The tight positioning of the external bumper results in the displacement of the internal bumper into the gastric wall. The type of tube material, tube size, and characteristics of the internal bumper of the PEG apparatus may also contribute to the development of BBS [3].

The classic presentation of BBS includes loss of PEG patency, leakage around the PEG site, and peristomal pain [14]. BBS is a clinical diagnosis, but an EGD or abdominal CT scan may be used for confirmation. Imaging also helps ascertain the depth and location of the displaced bumper [6]. BBS diagnosis is often delayed in non-verbal or asymptomatic patients, and provider education can lead to a prompt diagnosis. Due to concern for complications such as peritonitis and sepsis, immediate treatment of BBS is recommended even if the patient is asymptomatic.

The removal of the buried PEG depends on the peg set type and depth of the displaced internal bolster [12]. Endoscopic external extraction is the preferred modality for simple cases as it has lower morbidity and mortality [15]. Surgical intervention is effective but often invasive, therefore, an exploratory laparotomy should only be considered in gastro-peritoneal fistulas, peritonitis, or severe sepsis [16]. In case of abscesses, a patient would benefit from surgical drainage, broad-spectrum antibiotics, and local wound care. A replacement PEG may be placed at the same site or in a different site.

Clinicians should take various measures to reduce the likelihood of PEG-related complications. Proper care begins with the patient and nursing education for PEG tube use, emphasizing daily tube rotations. This practice allows for an early diagnosis of BBS, preventing further complications [1,3,12]. A study [17] reported a case of a 90-year-old female with a history of CVA who died from BBS-associated peritonitis and ileus due to delayed intervention. During PEG placement, endoscopists should not have gauze pads placed under the external bumper as that will predispose the patient to BBS. Gauze pads may be placed above the external bolster if necessary [4,6]. Additionally, when placing the PEG apparatus, the outer bolster must be approximately 0.5 to 1 cm from the abdominal wall to prevent excessive traction [6,8]. A greater distance is not encouraged due to the risk of content leakages [4]. Overall, an adequate positioning of the external bolster is the most important preventive measure against BBS.

## Conclusions

BBS is a rare yet serious complication of PEG but isolated cases of early onset have been reported in the literature. An early diagnosis is crucial to avoid further complications, and external endoscopic extraction is preferred for treatment. More research needs to be done to guide the prevention, diagnosis, and treatment of BBS.
